# Dietary Composition and Cardiovascular Risk: A Mediator or a Bystander?

**DOI:** 10.3390/nu10121912

**Published:** 2018-12-04

**Authors:** Emmanouil Korakas, George Dimitriadis, Athanasios Raptis, Vaia Lambadiari

**Affiliations:** Second Department of Internal Medicine and Research Institute, University General Hospital Attikon, 124 62 Haidari, Greece; mankor-th@hotmail.com (E.K.); gdimitr@med.uoa.gr (G.D.); atraptis@med.uoa.gr (A.R.)

**Keywords:** atherosclerosis, nutrition, nutrition interventions, chronic disease, microbiome

## Abstract

The role of nutrition in the pathogenesis of cardiovascular disease has long been debated. The established notion of the deleterious effects of fat is recently under question, with numerous studies demonstrating the benefits of low-carbohydrate, high-fat diets in terms of obesity, diabetes, dyslipidemia, and metabolic derangement. Monounsaturated and polyunsaturated fatty acids, especially *n*-3 PUFAs (polyunsaturated fatty acids), are the types of fat that favor metabolic markers and are key components of the Mediterranean Diet, which is considered an ideal dietary pattern with great cardioprotective effects. Except for macronutrients, however, micronutrients like polyphenols, carotenoids, and vitamins act on molecular pathways that affect oxidative stress, endothelial function, and lipid and glucose homeostasis. In relation to these metabolic markers, the human gut microbiome is constantly revealed, with its composition being altered by even small dietary changes and different microbial populations being associated with adverse cardiovascular outcomes, thus becoming the target for potential new treatment interventions. This review aims to present the most recent data concerning different dietary patterns at both the macro- and micronutrient level and their association with atherosclerosis, obesity, and other risk factors for cardiovascular disease.

## 1. Introduction

Cardiovascular disease (CVD) is the most common cause of death in Westernized societies, being responsible for one of every three deaths in the United States and one of every four deaths in Europe. The prevalence of obesity, type 2 diabetes mellitus (T2DM), and metabolic syndrome (MetS) may be the most common risk factors for it and has increased dramatically in recent years. According to the World Health Organization (WHO), the global prevalence of diabetes in adults has increased from 4.7% in 1980 to 8.5% in 2014, whereas worldwide obesity has almost doubled since the 1980s [1].

The role of nutrition in terms of reducing calorie intake and therefore body weight so as to prevent disease and lengthen life span has been well known since the 1930s, with the scientific society (along with popular belief) insisting specifically on the reduction of fat consumption. The Seven Countries Study was the first to demonstrate a clear link between the consumption of fat and the risk for cardiovascular events and set specific dietary rules for decades [2,3]. On the other hand, results from recent large randomized controlled trials (RCTs), like the PURE study, have raised serious questions as to what the ideal nutritional regimen is and to how each type of macronutrient, namely carbohydrates, proteins, and fats, affect body weight, glycaemic control, oxidative stress, inflammation markers, and other predisposing factors for CVD.

A notion that seems to prevail, however, is the beneficial role of the Mediterranean Diet (Med Diet) in the prevention of CVD. This benefit derives from the high consumption of fruits, vegetables, nuts, fish, poultry, and olive oil that this diet entails, together with a small quantity of red meat, emphasizing the importance of low-fat (LF) animal protein sources, complex plant-based carbohydrates, and high fiber content, as well as mono- and polyunsaturated lipids [4]. The majority of these foods contain an increased content of micronutrients that have anti-inflammatory capacities, decrease the oxidization of lipoproteins, and have a favorable effect on oxidative stress in general. Many studies have demonstrated the correlation between the Mediterranean Diet and every aspect of cardiovascular disease and its predisposing factors. The exact mechanism of how these micronutrients work is constantly unraveled. 

In addition, it is now known that gut microflora plays a significant role in human health. Changes in the composition of gut microbiota associated with disease, namely, dysbiosis, have been related to cardiometabolic derangement such as atherosclerosis and insulin resistance [5]. Hence, the effects of dietary patterns on the composition of intestinal microflora and the pathways through which different strains influence the main risk factors for CVD are in the spotlight and have emerged as possible treatment choices for the general population.

The objective of this review is to present and discuss the latest data regarding dietary patterns and their effect on cardiovascular disease through the compilation of different studies that have been published in recent years. The physiologic pathways through which macro- and micronutrients affect metabolic health are presented, along with the effect of dietary composition on gut microbiome and how this is connected to different cardiometabolic outcomes.

## 2. From Physiology to Disease: A Briefing on the Main CVD Risk Factors

A variety of factors have been associated with cardiovascular disease, such as metabolic syndrome, obesity, smoking, diabetes, hyperlipidemia, family history, hypertension, exercise, and more. The main risk factors and a summary of the pathophysiological mechanisms that lead to cardiovascular disease are described below.

### 2.1. Obesity

Obesity has risen sharply in the past decades. Worldwide, the proportion of adults with a body mass index (BMI) of 25 kg/m^2^ or greater increased between 1980 and 2013 from 28.8% to 36.9% in men and from 29.8% to 38% in women [6]. However, BMI estimates overall adiposity; it is also the distribution of body fat that counts. Fat accumulation in visceral adipose tissue leads to an influx of macrophages, the production of proinflammatory cytokines, dysregulated levels of adipokines, insulin resistance, and atherogenesis, as adiponectin can no longer suppress foam cell formation. In addition, accumulation of lipids within muscle cells and hepatocytes leads to further insulin resistance and abnormal glucose uptake [7]. It should be noted, however, that the optimal BMI with respect to mortality is yet to be defined, as even lean individuals can be insulin resistant, and obese persons can have no manifestations of metabolic syndrome. According to a meta-analysis [8], mortality was lowest at BMI 22.5–25.0 kg/m^2^. When adjusted for other traits, such as waist circumference or MetS, BMI is a much weaker predictor for CVD risk [9].

### 2.2. Insulin Resistance and Diabetes

As we mentioned above, insulin resistance can severely alter adipokine levels. It decreases the production of nitric oxide through inhibition of the phosphoinositol-3 kinase (PI3-K) pathway, thus resulting in endothelial dysfunction. In addition, it favors the secretion of endothelin-1, which leads to vasoconstriction and vascular smooth muscle cell (VSMC) proliferation. Insulin resistance also leads to reduced apoptosis of macrophage cells in atherosclerotic lesions, which eventually leads to plaque rupture [10]. Contrary to obesity, insulin resistance (and the consequent hyperinsulinemia) is an independent risk factor for cardiovascular disease, with a one-unit increase in the Homeostasis Model Assessment (HOMA-IR, which quantifies insulin resistance) being associated with a 5.4% increase in CVD risk [11].

### 2.3. Hyperlipidemia

Hyperlipidemia is characterized by the change in the lipid concentrations in plasma with the accumulation of one or more classes of lipoproteins. The major categories of lipoproteins consist of low-density lipoprotein (LDL), very-low-density lipoprotein (VLDL), high-density lipoprotein (HDL), and chylomicrons. They contain multiple proteins, called apolipoproteins, with the main one of LDL and VLDL being apoB-100, which is considered extremely atherogenic. VLDL also contains apoC and apoE. LDL has long been established as a major risk factor for CVD [12], and the mechanisms that lead to atherosclerosis are well described [13]. As LDL particles filter into the arterial intima, they are attacked by macrophages which are then transformed to foam cells. Accumulation of foam cells gives birth to fatty streaks. As foam cells die and smooth muscle cells produce fibrous connective tissue, the fatty streak turns into a fibrous plaque (fibroatheroma), which eventually becomes unstable and prone to rupture after years. Lp(a) is a complex lipoprotein that consists of an LDL and an apoB-100 particle linked to the plasminogen-like apolipoprotein (a) and is considered an even more atherogenic particle than LDL-C (low-density lipoprotein cholesterol). Lp(a) 3.5-fold higher than normal increases the risk for CVD events, particularly when LDL-C is more than 130 mg/dL [14], through mechanisms such as platelet aggregation, alteration of fibrin clot structure, and endothelial dysfunction. HDL-C (high-density lipoprotein cholesterol) contains the apolipoproteins apoA1 and apoA2 and is associated with favorable effects on CVD [15]. Hypertriglyceridemia occurs when the concentration of the triglyceride-rich VLDL particles is increased, along with a reduction in HDL-C. It is the result of the inhibition of lipoprotein lipase (LPL), which breaks down triglycerides so that free fatty acids (FAs) can be used by adipose tissue and muscles [16]. On fatty acid levels, the consumption of saturated fatty acids (SFA) increases inflammation by promoting the production of the proinflammatory type M2 macrophages and inhibiting the production of the anti-inflammatory type M1 macrophages. On the other hand, monounsaturated (MUFA) and polyunsaturated fatty acids (PUFA) ameliorate glucose tolerance and increase the levels of adiponectine, which is associated with higher HDL-C and lower triglycerides [17].

### 2.4. Oxidative Stress

Oxidative stress is a state of imbalance between oxidants and antioxidants in favor of the oxidants. Its association with vascular CVD risk has been established in many studies [18]. Oxidants, also called reactive oxygen species (ROS), include free radicals such as superoxide and peroxynitrite and nonradicals such as hydrogen peroxide. Their sources include enzymes like myeloperoxidases, uncoupled nitric oxide synthase (NOS), peroxidases and NADPH oxidase. Antioxidants include enzymes such as superoxide dismutase and catalase, nonenzyme molecules such as glutathione, and micronutrients [19]. Oxidative stress and inflammation are closely interrelated, and many studies have shown the association of inflammatory markers like high-sensitive CRP (hsCRP) and interleukin-6 (IL-6) with adverse metabolic outcomes [20]. The detrimental effects of oxidative stress in various pathways that are related to diabetes, obesity, endothelial dysfunction, atherosclerosis, hypertension, and generally every predisposing factor for cardiovascular disease are numerous, and their description is beyond the scope of this review. An excellent example of them is the oxidized LDL (ox-LDL), which is the result of the interaction of LDL particles with free radicals and is considered highly atherogenic. In endothelial cells, the main ox-LDL receptor is the LOX-1. TNF-α, adhesion molecules VCAM-1 and ICAM-1, ROS generated from NADPH oxidase, and other proinflammatory molecules increase the production and expression of LOX-1; on the contrary, the suppression of the inflammatory NF-kB pathway downregulates its expression, thus limiting the formation of foam cells and preventing atherosclerosis [21].

## 3. Low-Carbohydrate Diets vs. Low-Fat Diets: A Long-Standing Debate about the Role of Macronutrients on CVD

The Seven Countries Study [2], beginning in 1956 and originally published in 1978, was the first epidemiological study that examined the relationship between diet and cardiovascular disease in different populations and parts of the world. Its results consolidated the belief that higher fat intake (and especially high saturated fatty acids) was directly associated with higher prevalence of CVD, despite the existence of several randomized trial and studies throughout these decades that did not support this conclusion [22,23,24,25,26]. For example, the Nurses’ Health Study, originally established in 1976, showed an inverse association between total fat and total mortality, along with a higher risk of ischemic stroke due to a higher glycemic load [22]. Similar results were obtained from the Health Professionals Follow-up study (1985–2008) [23,24]. However, it was not until recently, with the publication of the Prospective Urban Rural Epidemiology (PURE) study in 2017, that the common belief about the adverse role of fat was again questioned.

In alignment with established notions, the 2016 European Society of Cardiology (ESC) guidelines has recommended a total fat intake of below 30%, of which 10% should only consist of saturated fats. Moreover, substitution of saturated fat with polyunsaturated fatty acids is strongly emphasized [27]. On top of that, in June 2017, the American Heart Association’s (AHA) presidential advisory on the lipid content of nutrition concluded that replacing saturated fat with polyunsaturated vegetable oil can reduce CVD by 30% [28,29]. 

The PURE study has questioned the guidance relating to fat consumption when it assessed the association between consumption of carbohydrate, total fat, and each type of fat with cardiovascular disease and total mortality. The study was a very large epidemiological one, with over 135,000 patients enrolled from 18 different countries of different income statuses across 5 continents. Patients were followed up for a median of 7.4 years. Outcomes included total mortality and major adverse cardiovascular events (MACEs) (CVD death, nonfatal MI, stroke, and heart failure). Higher carbohydrate intake was associated with an increased risk of total mortality but not with the risk of cardiovascular disease or cardiovascular disease mortality. On the contrary, fat intake was associated with a decreased risk of total mortality, regardless of the type of fat examined, and saturated fat intake was associated with a lower risk of stroke and total mortality [30]. A cross-sectional analysis of the PURE study also demonstrated that total fat intake, regardless of type, was associated with higher total cholesterol (TC) and LDL cholesterol, higher HDL cholesterol and apolipoprotein A1 (ApoA1), lower total:HDL cholesterol ratio, lower triglycerides:HDL ratio, lower triglycerides, and lower ApoB:ApoA1 ratio. The replacement of saturated fatty acids with carbohydrates or monounsaturated or polyunsaturated fats improved blood pressure, while surprisingly, the replacement of saturated fats with unsaturated fats had mixed results on the lipid profile of the patients [31]. 

In conclusion, the PURE study showed that total fat intake had at least neutral or even beneficial effects on cardiovascular health, while a high carbohydrate intake (>60% of diet) did not have a positive impact on atherosclerosis and CVD. It also proved that mortality is increased when refined carbohydrates replace saturated fat in an attempt to minimize fat consumption. Finally, the study emphasizes that the largest benefit in mortality is observed when refined carbohydrates are reduced and replaced by polyunsaturated fat [32]. Nonetheless, the study has drawn serious criticism in some points. The fact that most study participants came from low-income countries makes poverty and undernutrition a serious confounding factor regarding mortality. In addition, the reliability of dietary intake data has been questioned, as in China, for instance, other surveys have shown an average intake of around 30% of daily calories from fat, compared to 17.7% in PURE. Finally, the macronutrient analysis did not qualify the type of dietary carbohydrate as either simple (with low glycemic index) or complex (high in fiber and low glycemic index), which significantly differ in terms of incidence of cardiovascular events [33].

Low-carb, high-fat (LCHF) diets have proved to be at least as effective as low-fat, high-carb (LFHC) diets in weight loss [34,35,36]. A randomized controlled trial by Bazzano et al., in 2014 indicated that the LCHF diet group lost more weight (−5.3 kg compared to −1.8 kg) and had a 1.3% decrease in % body fat compared with a 0.3% gain in the LFHC group [37], a finding that is supported in other trials with both diabetic and nondiabetic patients [38,39,40,41]. Similarly, Shai et al., showed that after 24 months of follow-up, the LCHF diet group, despite being the only group that ate ad libitum (meaning, without energy restriction), experienced the greatest weight loss (−4.7 kg) compared with an LFHC group (−2.9 kg) and a Mediterranean Diet group (−4.4 kg) [42]. On the other hand, a meta-analysis by Bueno et al., showed no significant difference in weight loss between LCHF and LFHC diets [43]. Hjorth et al., observed that prediabetic and diabetic individuals lost more weight when they were on an LCHF diet, whereas individuals with normoglycemia had better results with a low-fat diet [44]. 

Many studies have also shown a beneficial impact of LCHF diets on lipid markers. LCHF diets have proved more effective than LFHC ones at lowering triglycerides and ApoB—a risk predictor for coronary artery disease—and increasing HDL cholesterol [45,46,47]. On the other hand, a common argument against LCHF diets is the increase in LDL cholesterol that follows higher fat intake [48]. Tay et al., showed an increase of total cholesterol (+0.7 vs. +0.1 mmol/L) and LDL-C (+0.6 vs. +0.1 mmol/l) in the LCHF group [49], while Hu et al., also documented higher decrease of TC and LDL-C in the LFHC groups [50]. However, what should be taken into account is that the increase in HDL-C by itself is considered highly cardioprotective [51], and that the differences in LDL-C concentrations should be evaluated along with the changes in the LDL particles (the small ones are more atherogenic) [52]. Morgan et al., documented that although the subject’s initial LDL-C phenotype affected the changes in the size of LDL particles, the LCHF diets were associated with greater and less dense LDL particles in general [53]. However, it is true that the increase in LDL remains a serious concern regarding the low-carbohydrate diets which needs to be assessed in more studies in the future.

Finally, a recent study published in the Lancet Public Health has added fuel to the fire, enrolling 15,428 adults from the Atherosclerosis Risk in Communities (ARIC) study, who completed a dietary questionnaire. The researchers investigated the association between the percentage of energy from carbohydrate intake and all-cause mortality. The results suggested that both high- and low-carbohydrate diets were associated with increased mortality, with an optimal carb intake of 50–55%. Higher mortality was observed when carbohydrates were substituted by animal-derived protein and fat, while lower mortality was achieved when protein and fat were derived from plant sources and whole grains [54]. The paper made headlines around the world stating that a low-carb diet will shorten life. However, a lot of criticism has risen, mainly due to the observational nature of the study, the possible confounders like the whole unhealthy lifestyle (i.e., alcohol consumption, environmental toxins, smoking) that could interfere with the results apart from the diet, and the weak associations found that actually are just epidemiological observations that contradict strong data so far [55].

## 4. Choosing the Right Type of Fat: A Comparison of Fatty Acids

The consumption of different types of fatty acids causes different metabolic changes. As T2DM is a leading predisposing factor for CVD, the effects of fatty acids on insulin metabolism are of profound significance. Many human studies have shown an association between diets rich in saturated fatty acids (SFAs) and poor in polyunsaturated fatty acids (PUFAs) with insulin resistance [56,57,58,59]. Mullner et al., showed that the supplementation of PUFA-rich plants in the daily dietary regimen of diabetic and nondiabetic individuals resulted in a significant reduction of HbA1c [60], and similar were the results in a study by Lee et al., where subjects with T2DM or metabolic syndrome were provided with capsules of fish oil compared to other types of oil. In diabetics, the supplementation of 3 g of alfa-linolenic acid (ALA) per day resulted in improved insulin sensitivity, as did the supplementation of conjugated linoleic acid (CLA), a group of isomers of linoleic acid rich in PUFAs, in obese children. Among the SFAs, palmitic acid is thought to have the most detrimental effect on pancreatic β-cell function [61]. Koska et al., showed that a diet rich in SFAs resulted in higher glucose and insulin concentration after 24 h compared to a diet where saturated fats comprised only 5% of total calories ingested [62]. As for animal studies, Malinska et al., found that the supplementation of hypertriglyceridemia-induced dyslipidemic rats with CLA resulted in enhanced insulin sensitivity [63]. An in vitro study in mice showed that the administration of palmitate induced insulin resistance in cardiovascular cells, contrary to the favorable effect of oleate [64]. MUFAs also exhibit a beneficial role regarding glucose metabolism, being associated with an important decrease in HbA1c levels, while stimulating GLP-1 secretion, at least in animal models [65].

Regarding dyslipidemia, SFAs have a strong atherogenic effect due to the reduction in the expression of the LDL receptor gene, thus increasing the concentration of LDL in circulation [66]. On the contrary, PUFAs are considered to ameliorate lipid markers, with *n*-3 PUFA consumption resulting in reduction of plasma triacylglycerols and ApoB-100, which in turn reduces the concentration of VLDL and LDL [67]. Docosahexanoic acid (DHA)—the main *n*-3 PUFA together with eicosapentanoic acid (EPA)—improves cellular membrane fluids, an action that results in the removal of lipids and inflammatory agents from the arterial wall [68], while both these *n*-3 PUFAs have been found to decrease the expression of sterol regulatory element-binding protein-1c (SREBP-1c), hence reducing the production of VLDL and triacylglycerols (TAGs) [69]. EPA and DHA have also been shown to increase HDL when supplemented in various daily doses in humans, which, however, exceed the normal daily intake [70,71]. In an RCT where patients with impaired glucose metabolism and coronary artery disease were supplemented with 1800 mg/day EPA for six months, postprandial hypertriglyceridemia, hyperglycemia, and insulin secretion were ameliorated compared to placebo [72]. The REDUCE-IT trial showed that the supplementation of 2 g per day of icosapent ethyl, a highly purified eicosapentaenoic acid ethyl ester, led to a reduction in ischemic events, including cardiovascular death, compared to placebo [73]. Unlike these, *n*-6 PUFAs have not been found to have such beneficial effects on lipid markers universally, according to several studies with rodents [74,75]. Regarding MUFAs, the data are more limited, but in vivo studies in Wistar rats like those of Macri et al., and Alsina et al., found that olive oil and fish oil, rich in MUFAs, were associated with a decrease in TC and LDL and better fat distribution in tissues [76,77]. Contrary to the aforementioned findings, however, the VITAL trial, an RCT which examined the results of the supplementation of both vitamin D_3_ (at a dose of 2000 IU per day) and *n*-3 fatty acids (at a dose of 1 g per day) among adults in the United States, showed no statistically significant difference in the incidence of cardiovascular events compared to the placebo group. [78]

Fatty acids also intervene with inflammation markers which provoke oxidative stress. A study in adult individuals which compared four diets with different FA composition found that n-3 PUFAs decreased postprandial endotoxin levels, unlike the n-6 ones [79]. Matsumoto et al., showed that, in ApoE−/− mice, supplementation of EPA resulted in amelioration of atherosclerotic lesions with less macrophage infiltration and more collagen concentration [80]. Similarly, Simopoulos et al., showed that n-6 PUFAs increase cellular triglycerides and the permeability of the membrane, thus increasing adipose tissue fat, which has strong inflammatory capacities [81]. In another study in obese adults, a diet rich in palmitic acid, compared to a diet rich in oleic acid, decreased the concentrations of the inflammatory cytokines IL-1B, IL-10, IL-18, and TNF-α [82]. In agreement with these findings in human studies, Moya-Perez et al., showed that high-fat diets increase the infiltration of lymphocytes B in rats, which in turn increases the release of proinflammatory macrophages, IL-8, and IFN-γ cytokines [83]. A higher size of adipocyte cells has also been observed with high-fat diets in rats, leading to an increase of NF-γ, TNF-α, and other inflammatory markers [84].

## 5. Mediterranean Diet: The Golden Standard against Cardiovascular Disease?

Mediterranean Diet is the dietary pattern of countries around the Mediterranean Sea. Despite the relatively high fat intake (35–45%), it has long been considered to be one of the most cardioprotective diets because of its abundance in unsaturated fats and other micronutrients with a beneficial impact on CVD risk factors. It comprises a high intake of olive oil, fruits, vegetables, legumes, nuts, and cereals; a moderate intake of fish and poultry; low consumption of red meat and sweets; and a moderate consumption of wine, mainly with meals [4]. The Lyon Diet Heart Study was the first secondary prevention trial that found that adherence to this dietary pattern was associated with a reduced rate of recurrence after a first myocardial infarction [85], and since then, a number of observational studies or randomized controlled trials have favored these cardioprotective effects [86,87,88,89,90].

One landmark study is the PREDIMED (Prevención con Dieta Mediterránea) study, which took place in Spain. Seven thousand four hundred and forty-seven participants at high cardiovascular risk, but with no cardiovascular disease at enrollment, were divided in three groups according to the dietary pattern that they had to follow: a Mediterranean Diet supplemented with extra-virgin olive oil, a Mediterranean Diet supplemented with nuts, and a control low-fat intake group. The median follow-up was about 4.8 years. The study found that the groups adherent to the Mediterranean Diet had a relative risk reduction of about 30% for major cardiovascular events, namely, myocardial infarction, stroke, and death from cardiovascular causes [91]. In addition, a cross-sectional analysis of a subgroup of the participants in the PREDIMED by Salas-Salvadó et al., demonstrated that after a follow-up of four years, diabetes incidence was 10.1%, 11%, and 17.9% in the extra-virgin oil group, the nuts group, and the control group, respectively, while when the two Med Diet groups were compared together with the control group, diabetes incidence was reduced by 52% in total [92]. Decreases in blood pressure and the total cholesterol:HDL-C ratio and waist circumference were also pointed out.

However, in June, 2017, concerns were raised about the implausibility of the distribution of baseline data in the original primary publication of PREDIMED. After an investigation, the New England Journal of Medicine retracted and republished the primary PREDIMED paper with a modified analysis to account for protocol deviations and departures from individual randomization in a subsample of patients during the trial [93].

Again in favor of the Mediterranean Diet, Tektonidis et al., found that, after a 10-year follow-up, higher adherence to a Mediterranean Diet was associated with a statistically significant reduction of 26%, 21%, and 22% risk for myocardial infarction, heart failure, and ischemic stroke, respectively, while no association was found regarding the risk of hemorrhagic stroke [94]. These results were consistent with results from another cohort study of 30,000 participants in the United States [95]. In a report from the CORDIOPREV study, the endothelial function of 805 participants was measured using ultrasonography of the brachial artery to calculate flow mediated vasodilatation before and after 1.5 years of adherence to a Mediterranean dietary pattern or low-fat diet. Results found that Med Diet improved the flow-mediated dilatation (FMD) (and, thus, endothelial function) in patients with diabetes and prediabetes [96]. The CARDIA study showed that the consumption of fruits and vegetables at a rate of seven to nine servings per day in early adulthood had an inverse association with the prevalence of coronary artery calcium after 20 years of follow-up [97]. In the Multiethnic Study of Atherosclerosis (MESA), a higher Med Diet score was associated with better left ventricular (LV) structure and function, which was attributed to a higher LV volume, higher ejection fraction, and higher stroke volume, despite some increase in LV mass [98]. In addition, the higher Med Diet score was associated with less visceral and peripheral fat (but not subcutaneous fat) and less hepatic steatosis [99]. Finally, the recently published EPIC study, which used nine dietary components of the Med Diet (score 0–18), found an inverse association of Med Diet with risk of developing T2DM [100]. 

The positive effects of Mediterranean Diet cannot be attributed specifically to every single constituent of it. It seems that it is the combination of the different nutrients that is responsible for its metabolic benefits. Olive oil, having oleic acid, a MUFA, as its major component, is highly effective in decreasing the oxidization of LDL [101]. Wine’s favorable effects on HDL-C, waist circumference, blood pressure, and hyperglycemia have been shown in several studies [102,103], in accordance with the PREDIMED study, where moderate wine intake was associated with a 44% reduction in the prevalence of MetS compared with abstainers. Studies on fish, and especially fatty fish which are rich in n-3 PUFAs, have shown a positive effect on body weight, blood pressure, glucose homeostasis, as well as lipid markers, an effect that possibly has to do with changes in HDL particles [104,105,106]. A meta-analysis of six prospective studies showed that nut consumption protects from coronary heart disease and reduces the risk for development of T2DM [107], while another meta-analysis of 49 RCTs reported improvements in at least one criterion of MetS with higher nut consumption [108]. Finally, dairy products, and especially low-fat ones, have been associated with reduced risk of MetS [109], a finding that was repeated in a recent meta-analysis of 2016, where high consumption led to a 15% reduction in risk [110]. Other meta-analyses have reported an inverse association of dairy consumption with T2DM risk [111,112,113]. However, all these data refer mainly to low-fat products and yoghurt, with cheese (especially yellow cheese) having rather controversial effects on metabolic health. Interestingly, recent data imply that dairy consumption and in particular odd-chain saturated fatty acids, like pentadecanoic acid (C15:0) and heptadecanoic acid (17:0), may have a beneficial effect on beta-cell function and in diabetes prevention [114]. 

In recent years, some alternative dietary regimens to the Mediterranean Diet have emerged. The Dietary Approaches to Stop Hypertension (DASH) Diet has been created by the US-based National Heart, Lung, and Blood Institute of Health, and enjoys high approval both in the United States and worldwide. It consists of fruits, vegetables, whole grains, poultry, fish, nuts, and minimal consumption of red meat, sweets, and sugar-sweetened beverages. The main differences with the Med Diet have to do with the higher content in fat in the latter, through the consumption of olive oil and the higher consumption of dairy products in the former, along with a limitation in sodium intake. The DASH Diet has proved to be effective mainly in decreasing blood pressure [115,116], and a subanalysis in the MESA study has also shown favorable effects on left ventricular function in terms of a higher end-diastolic volume and a (marginally significant) increase in ejection fraction [117]. However, data regarding the association of the DASH Diet and MetS are rather scarce. Another dietary pattern that has recently been proposed is the Nordic Diet, due to poor adherence of people in Nordic countries to the Mediterranean Diet. It includes high intake of fish, apples, cabbages, root vegetables, pears, and rapeseed oil. In an RCT of 200 individuals with MetS, adherence to the Nordic diet resulted in amelioration of lipid markers [118], while in a Danish study by Hansen et al., high adherence to the Nordic Diet was associated with a 14% lower risk for ischemic stroke compared to low adherence, according to a Healthy Nordic Food Index score [119]. Rapeseed oil is probably the most highlighted component of this diet, as it is an excellent source of fat due to its high concentration in mono- and polyunsaturated fatty acids and to its lowest omega-6:omega-3 ratio of the commonly used vegetable oils. 

In accordance with the aforementioned results, a recent systematic review of 14 studies and a total of 188,470 participants compared the Mediterranean, DASH, vegetarian, and Paleolithic diets for primary prevention of heart failure and demonstrated that both the Mediterranean and DASH diets exert a protective effect on the incidence of heart failure and of cardiac function parameters’ deterioration rate [120]. Another recent systematic review included 12 studies and 4201 participants and examined the effect of several dietary patterns on the secondary prevention of heart failure. The results favored the DASH Diet as the beneficiary for the secondary prevention of heart failure, with the Mediterranean Diet exhibiting a positive but less robust correlation with some factors of secondary prevention [121]. 

## 6. Micronutrients: Functional Patterns and Benefits in Metabolism and Cardiovascular Disease

Micronutrients are essential elements that are required in very small quantities in the human organism, estimated at milligrams per day, and have a vast array of biochemical functions that regulate metabolism and homeostasis in general. As diabetes, atherosclerosis, and metabolic syndrome are associated with chronic systemic inflammation and oxidative stress, micronutrients have drawn popular interest due to their antioxidant capacities. By intervening in various molecular pathways, they are potent scavengers of ROS or they can limit the generation of free radicals and inflammatory agents [122]. The main micronutrients and their effect on metabolic health are presented below.

### 6.1. Polyphenols

Polyphenols are the most abundant antioxidants in diet and, in general, can be found mainly in fruits, vegetables, green tea, red wine, nuts, spices, and extra-virgin olive oil. Flavonoids are the most common polyphenols, which in turn are divided in six subclasses: flavanols, flavones, flavanones, anthocyanins, flavonols, and isoflavones. Polyphenols have been associated with an inverse relationship with T2DM, as they inhibit α-glucosidase and α-amylase and increase GIP and GLP-1 peptides, thus resulting in a better glucose homeostasis, as it was shown both in a small study in which humans who consumed caffeinated and decaffeinated coffee in a usual dose were compared [123], and in diabetic mice where high doses of resveratrol (a polyphenol analyzed below) were supplemented for weeks [124,125]. Phenols like tannic and chlorogenic acids can interact with SGLT-1 and SGLT-2 co-transporters and thus inhibit glucose absorption [126]. Beneficial effects have also been mentioned regarding stroke. Diets rich in anthocyanins in rats have been associated with better neuroprotection after stroke and reduced cerebral ischemia and oxidative stress in both human and animal models [127,128]. Epigallocatechin-3-gallate (EGCG), a polyphenol in green tea, also proved to have neuroprotective impact in cases of cerebral ischemia in rats in vivo [129], a finding that applied also to quercetin, which protected the integrity of the blood-brain barrier in rats where it was administered intraperitoneally by reducing levels of matrix metallopeptidase 9 (MMP-9) [130]. 

Resveratrol is a flavonoid that is mainly found in red wine. In some in vivo studies in rats, resveratrol has been found to reduce plasma triglycerides and LDL-C and to increase HDL-C [131]. It decreases the oxidization of LDL particles and increases the expression of LDL receptors in hepatocytes in vitro [132]. By activating SIRT-1, eNOS, and Nrf2 pathways, it decreases the concentration of TNF-α, a major inflammatory cytokine [133]. It also decreases the expression of adhesion molecules ICAM-1 and VCAM-1 via inhibition of the NF-κB pathway activation [134] and reduces the formation of foam cells via inhibition of NADPH oxidase-1 in mouse macrophages [135]. The cumulative result of the aforementioned actions is a reduction of vascular inflammation and enhanced endothelial function. In studies with diabetic patients [136], healthy obese men [137], and healthy adult smokers [138], resveratrol showed beneficial effects on the lipid profile of the subjects, yet this result was achieved through the supplementation of high doses of resveratrol (250–1000, 150, and 500 mg/day, respectively), which cannot be achieved by normal daily food intake. As for hypertension, resveratrol increases the bioavailability of nitric oxide (NO) in human umbilical vein endothelial cells (HUVECs) in vitro [139] as well as its production in animal models [140], while in clinical studies, antihypertensive results have been shown only in high concentrations [141]. In some studies in rodents, pretreatment with resveratrol after a myocardial infarction decreased infarct size and arrhythmias [142,143]. In general, the low availability of resveratrol due to its rapid metabolism seems to limit its clinical potential.

Luteolin is a flavonoid present in many medicinal plants and in vegetables such as celery and parsley. Many studies have demonstrated its antioxidant and anti-inflammatory capacities [144,145]. It inhibits oxidization of LDL and expression of VCAM-1, while it decreases plasma lipids and benefits vascular dilatation through enhanced expression of eNOS gene [146,147]. These results were demonstrated both in in vivo and in vitro studies with humans or rodents. The main concern regarding many of these findings was that they took place in pharmacological (>10 μΜ) rather than physiological (<2 μΜ) concentrations of luteolin. However, a recent study in mice and in human endothelial cells by Zhenquan et al., showed that, even in low doses, luteolin inhibited TNF-α-induced binding of monocytes to endothelial cells, activation of NF-κB pathway signaling, vascular inflammation, and changes in the intima layer of the aorta, thus confirming its cardioprotective role regardless of its levels [148].

Quercetin is a flavonol that can be found in many vegetables, like red onions, capers, and kale. As mentioned above, it has a beneficial role in stroke and also improves lipidemic profile. In vitro studies have shown that quercetin increases the expression of PPAR-γ receptors and ATP-binding cassette transporter (ABCA1), which leads to reduced formation of foam cells [149]. It also reduces oxidative stress. In ApoE-knockout mice, quercetin reduced hydrogen peroxide and leukotrien B4 in vessels and increased endothelial nitric oxide synthase (eNOS) [150]. Kim et al., found that quercetin, through activating LKB1-AMPK signaling pathway, inhibited myosin light chain kinase (MLCK) and phosphorylated myosin light chain (p-MLC), hence inhibiting vascular smooth muscle cell (VSMC) contraction in rats [151]. In vitro studies in mice have indicated that quercetin promotes glucose transport within adipose and muscle cells through GLUT4 transporters’ translocation and the AMP-activated protein kinase pathway, favoring normoglycemia [152]. Though it is true that most studies regarding quercetin have been conducted in animal models, there are also human studies that support its beneficial role. Obese subjects aged 25–65 years with metabolic syndrome were randomized to receive 150 mg quercetin/day for six-week treatment times. Compared to placebo, quercetin reduced systolic blood pressure by 2.6 mmHg in the entire group, by 2.9 mmHg in the subgroup of hypertensive patients, and by 3.7 mmHg in the subgroup of younger adults (25–50-years old), as well as reducing the total concentration of HDL-C and oxidized LDL [153]. In another study by Lee et al. [154], quercetin-rich supplements based on onion peel extract given to adult subjects for 10 weeks significantly reduced LDL-C, total cholesterol, systolic and diastolic blood pressure, and increased HDL-C. An RCT in humans showed that 730 mg of quercetin/day for four weeks decreased systolic and diastolic blood pressure in stage 1 hypertensive patients [155]. Another trial in diabetic women indicated that 500 mg of quercetin/day for 10 weeks reduced systolic blood pressure [156]. A large meta-analysis by Serban et al., on RCTs about the effect of quercetin on blood pressure reported that quercetin achieved a statistically significant decrease of blood pressure only in doses of >500 mg/day [157]. As it is clear, quercetin’s beneficial effects take place only in pharmacological doses that highly exceed normal daily food intake, taking into account that in the United States, for example, the daily intake of quercetin is about 10 mg.

Curcumin is a flavonoid that is mainly found in turmeric, curry spice, and ginger. Its role in improving oxidative stress has been marked in many studies in animal models, all of which attribute this capacity to the inhibition of the TLR4 signaling pathway [158,159]. Studies in ApoE−/− mice indicate that supplementation with curcumin leads to less macrophage infiltration in atherosclerosis plaque, reduced aortic NF-κB activation, reduced levels of IL-1B and TNF-α, and reduced expression of the adhesion molecules ICAM-1 and VCAM-1 [160]. 

### 6.2. Carotenoids

Carotenoids are lipophylic antioxidants that are found in plants and some photosynthetic bacteria and fungi. They are classified into carotenes and xanthophylls. Carotenes include beta-carotene and lycopene, and xanthophylls include lutein, fucoxanthin, zeaxanthin, canthaxanthin, astaxanthin, beta-criptoxanthin, and capsorubin [161]. 

Astaxanthin is found in plankton, fish (mainly salmon—200 g of salmon contains about 0.5–1.25 mg of astaxanthin), and other seafood. It is a strong free radical scavenger and decreases LDL-C and triglycerides, increases HDL-C, and ameliorates inflammation markers both in human and animal models [162,163]. An RCT by Yoshida et al., in nonobese humans aged 20–65 years reported that the supplementation of astaxanthin (0, 6, 12, and 18 mg/day, respectively, in each group) for 12 weeks improved HDL-C and TG, as well as adiponectin levels [164]. Similarly, Iwamoto et al., reported improvements in LDL-C levels after supplementation with astaxanthin (from 1.8 to 21.6 mg/day) for two weeks in healthy volunteers [165]. In another study by Hussein et al., astaxanthin lowered blood pressure in hypertensive rats, an effect that was attributed to modulation of nitric oxide levels [166].

Lycopene is found mainly in tomatoes, watermelons, and red grapefruits. Many studies have associated low plasma levels of lycopene with early carotid atherosclerotic lesions [167]. The Rotterdam Study, a prospective cohort study on adults aged 55 years or older, revealed an inverse association between lycopene and calcified plaques in the abdominal aorta, a result that was more evident in current and former smokers [168], whereas another report by Rissanen et al., in middle-aged adults supports that lycopene has a beneficial impact on carotid intima media thickness and cardiovascular events in general, at least in men [169]. It increases HDL-C, lowers triacylglycerols and ox-LDL [170], and contributes to vasodilatation through modulation of NO. Its relationship with T2DM risk remains controversial.

Lutein is found mainly in dark green vegetables such as spinach, parsley, and broccoli. It is a ROS scavenger and inhibits the NF-κB pathway [171]. It has been proved to decrease TNF-α, IL-6, PGE2, and oxidative stress in general, at least in rodents for which high pharmacological doses of lutein were administered [172]. A diet with high lutein intake is beneficial for atherosclerosis and arterial stiffness, as large studies like the ARIC [173] and the CUDAS [174] have demonstrated.

Beta-carotene is found primarily in carrots, tomatoes, and spinach. It decreases oxidization of LDL [175] and inhibits the NF-κB-induced expression of adhesion molecules in vitro [176]. It also reduces non-HDL cholesterol levels and the expression of IL-1a and VCAM-1 in mice in vitro [177]. In mature adipocytes, beta-carotene is metabolized to retinoic acid, which decreases the expression of PPAR-α and CCAAT/enhancer-binding protein, thus reducing the lipid content of adipocytes [178]. Finally, there seems to exist an inverse association between T2DM and consumption of beta-carotene with diet [179].

### 6.3. Trace Elements

Although a number of epidemiological studies have marked a selenium deficiency in people with diabetes, RCTs have not managed to prove that supplementation with selenium decreases the risk for T2DM and cardiovascular mortality at all [180]. In a recent systematic review, Sarmento et al., indicated that in diabetic patients, low levels of selenium were associated with an increased risk for CVD, but clearly pointed out the need for more studies in humans to solidify these results [181]. On the contrary, data that show that zinc deficiency is associated with vascular diseases are more stable. Low levels of zinc cause increased oxidative stress in endothelial cells in vitro [182], and they have been associated with increased cardiovascular events in diabetic patients. In a recent study in diabetic mice by Miao et al., supplementation of zinc (5 mg ZnSO_4_/kg per day for three months) reduced aortic tunica media thickness and collagen accumulation, apoptotic cell death and expression of inflammatory markers VCAM-1 and PAI-1, and increased the expression of Nrf2 and metallothioneins in the aorta, both of which have strong antioxidant properties [183]. It should be noted, however, that this dose does not represent daily human intake, which is about 0.07–0.23 mg/kg/day.

### 6.4. Vitamins

The data regarding the cardioprotective effects of B-complex vitamins is controversial. However, according to some studies in humans, high intakes of B6, B12, and folic acid may be beneficial for both ischemic and hemorrhagic stroke prevention, possibly due to the reduction of plasma homocysteine levels, and a diet rich in B vitamins can reduce poststroke functional decline [184,185]. Chambers et al., found that supplementation of folic acid and B12 at a dose of 5 and 1 mg per day, respectively, for eight weeks significantly improved endothelial dilatation [186] (the daily intake for adults in the United States is about 500 mcg/day for folic acid and 3.4 mcg/day for B12). Coenzyme Q10 or ubiquinone, a lipid-soluble benzoquinone that acts as a vitamin, has also been associated with favorable cardiovascular effects when supplemented to the normal diet [187,188]. Q10 also serves as an antioxidant, reducing lipid peroxidation and increasing the levels of antioxidant enzymes catalase (CAT) and superoxide dismutase (SOD) in vitro, after a daily treatment with 150 mg for 12 weeks, when its normal daily intake is no more than 3–5 mg [189]. Vitamins C (mainly found in citrus fruits) and E (mainly found in seeds and vegetable oil) are associated with a reduction in coronary artery disease (Law et al.) [190] and have antioxidant and anti-inflammatory properties, but their supplementation beyond the usual dietary intake does not have any proven benefits for humans [191,192]. Vitamin E is also considered to have favorable effects on glucose homeostasis, as it inhibits the formation of advanced glycosylation end products, reduces HbA1c, and prevents oxidative stress in pancreatic β-cells [193,194]. As for vitamin D, the recently published VITAL study (described above) showed that supplementation with vitamin D did not result in a lower incidence of cardiovascular events [195].

## 7. Gut Microbiota, Diet, and Cardiovascular Disease: A Recently Discovered Field

In recent years, a lot of interest has been drawn to the role of gut microbiota in human health. The gut microbiome encompasses 10^14^ microorganisms. The bacterial population is composed mainly of five phyla: *Firmicutes* (60–65%), *Bacteroidetes* (20–25%), *Proteobacteria* (5–10%), *Actinobacteria* (about 3%), and *Cerrucomicrobia* [196]. These microorganisms participate in the food digestion process through two catabolic pathways. In the saccharolytic pathway, gut microbiota break down sugars and produce most of the short-chain fatty acids (SCFAs), whose byproducts, mainly acetate, butyrate and propionate, are a major source of energy for intestinal epithelium. More specifically, SCFAs signal to the host through four different pathways at least. First, they are an energy substrate for colonocytes. Second, butyrate and acetate act as histone deacetylase inhibitors. Third, propionate can induce intestinal gluconeogenesis, with a favorable effect on glucose tolerance, and fourth, they activate G-protein-coupled receptors like GPR41 and GPR43, triggering the release of GLP-1, which plays a crucial role in glucose homeostasis. Fatty acid oxidation is activated by SCFAs, while de novo synthesis and lipolysis are inhibited, leading to a decreased concentration of free fatty acids in plasma and weight loss [197,198,199]. In the proteolytic pathway, protein fermentation takes place, along with SCFA formation and the production of many other metabolites, some of which are toxic for the host [200]. Primary bile acids, cholic acid (CA), and chenodeoxycholic acid (CDCA) are metabolized into the secondary bile acids deoxycholic acid (DCA) and lithocholic acid (LCA), which signal to the host through G-protein-coupled receptor 1 (also called TGR5) and the bile acid receptor FXR, both of which affect metabolism [201]. On the other hand, microbes produce endotoxins (also called lipopolysaccharides) which promote systemic inflammation and subsequently lead to insulin resistance. Another important pathway is that of the TMAO production. As microbes metabolize phosphatidylcholine and L-carnitine, they produce trimethylamine (TMA), which is then oxidized in the liver to trimethylamine N-oxide (TMAO) that has been associated with atherosclerosis both in humans [202] and mice. It is also well known that that the microbiota contribute both to local intestinal and systemic immunity through their effects on toll-like receptor (TLR) expression, macrophages, T cells, antibodies, and other mechanisms [203,204]. Changes in the composition of the gut microbiota have been associated with dietary changes, even short and acute ones, and have been linked to metabolic syndrome and cardiovascular disease in many aspects that are analyzed below.

### 7.1. Diet and Gut Microbiota

(a) Proteins. Many studies have shown associations between protein intake and gut microbial diversity [205,206]. In small human studies, whey and pea protein increased the population of the beneficial *Bifidobacterium* and *Lactobacillus* species, together with levels of SCFAs [207,208,209]. On the contrary, animal protein increases anaerobes such as *Bacteroides* and *Alistipes* [210]. Obese men were given a high protein/low carbohydrate diet for four weeks, which eventually reduced the concentrations of *Roseburia* and *Eubacterium rectale* populations in fecal samples with a concomitant reduction in the concentration of butyrate in feces [211], a finding that was confirmed in another study by De Filippo et al. This study compared the fecal microbiota of European children on a high-protein diet to those of children in Burkina Faso, where a high-fiber diet is followed, and demonstrated a lower concentration of fecal SCFAs in the first group [212]. The high intake of red meat as a source of protein has also been related to increased levels of trimethylamine-n-oxide (TMAO), which is an oxidation product of the microbial metabolite TMA and has been shown to have detrimental effects on atherogenesis [213].

(b) Fats. High fat intake increases the population of *Bacteroides*, *Clostridiales*, and *Enterobacteriales* and decreases the counts of the beneficial *Lactobacillus intestinalis* species, as studies in rats have indicated [214,215]. On the contrary, a low-fat diet increases fecal levels of *Bifidobacterium*, with positive effects on blood glucose homeostasis and total cholesterol levels. The type of fat consumed is also important. Mice supplemented with fish oil for 11 weeks had increased counts of *Lactobacillus* and *Actinobacteria*, while mice supplemented on lard oil had increased counts of *Bacteroides* and *Bilophila*, which led to increased TLR activation, impaired insulin sensitivity, and white adipose tissue inflammation [216]. Diets with n-6 PUFAs increase the number of *Firmicutes*, *Actinobacteria*, and *Proteobacteria* strains and decrease that of *Bifidobacteria*, which are associated with chronic inflammation and metabolic syndrome, mediated by an increase of lipopolysaccharides (LPS) [217].

(c) Carbohydrates. Digestible carbohydrates, which include starch and sugars (glucose, fructose, sucrose, and lactose) increase the number of *Bifidobacteria* strains and decrease that of *Bacteroides* in studies in vitro [218]. A study in which infants with cow’s milk allergy received lactose for two months showed that lactose increased SCFAs in fecal samples compared to the placebo group [219]. Nondigestible carbohydrates such as fiber act as prebiotics—nondigestible dietary components that selectively stimulate the growth and/or activity of certain microorganisms, thus affecting the host’s health beneficially [220]. In two studies where adult subjects received a whole-grain breakfast contrary to the placebo group, prebiotics increased *Bifidobacteria* and *Lactobacilli* strains and had favorable effects on glucose homeostasis and inflammation markers, such as IL-6 and IL-10 [221,222].

(d) Dairy products. Milk and yoghurt are rich in lactic acid bacteria and are considered probiotics (live microorganisms that, in appropriate amounts, benefit the host’s health) [223]. They increase *Bifidobacteria* and *Lactobacilli* population, as has been indicated in various human studies [224,225,226]. In an RCT where overweight adults were supplemented with probiotics, an enhancement in lipid markers and hsCRP was demonstrated [227]. Hanie et al., found that the administration of probiotic yoghurt to diabetic patients decreased fasting blood glucose and HbA1c compared with the control group after a follow-up period of six weeks [228].

### 7.2. Gut Microbiota and CVD

(a) Atherosclerosis and coronary artery disease: The bacteria that were found in atherosclerotic plaques were also found in the gut and oral cavity of the same individuals in a number of studies, implying that these bacteria could be a source of plaque colonization [229,230]. After sequencing of the gut metagenome in patients with unstable plaques, differences in microbiota composition were observed, associated with lower fecal levels of the genus *Roseburiam* and higher levels of the genus *Collinsella*, together with increased oxidative stress [231]. In rodents, increased TMAO levels were associated with an enhanced aortic atherosclerotic plaque [232], and similar findings have emerged in humans. In a large cohort study with over 4000 patients undergoing elective coronary angiography, it was reported that high TMAO levels were associated with higher incidence of CVD events over a three-year follow-up period [202]. Such results have been confirmed in other trials with over 1800 patients undergoing elective coronary angiography, where TMAO levels were associated with higher atherosclerotic plaque size, vulnerable atherosclerotic plaque, and higher all-cause mortality [233,234].

(b) Dyslipidemia: As mentioned above, gut microbiota produce secondary bile acids, which can alter both hepatic and systemic lipid metabolism through FXR and GPR131 [235,236]. Increased TMAO levels have been associated with deleterious effects on the lipid profile, affecting mechanisms such as reverse cholesterol transport and sterol metabolism, as studies in obese mice which underwent vertical sleeve gastrectomy indicated [237].

(c) Heart failure: The gut hypothesis in heart failure claims that the impairment in hemodynamics leads to intestinal mucosal ischemia and edema and increases bacterial translocation and production of endotoxins that exacerbate the already-existing oxidative stress and systemic inflammation associated with heart failure [238]. A confirmation of this hypothesis was provided by Pacini et al., who found increased concentrations of microorganisms in feces of patients with heart failure, which were associated with increased membrane permeability [239]. In a study by Tang et al., the relationship between fasting plasma TMAO and all-cause mortality over a five-year period in stable patients with heart failure compared to healthy controls was examined. Patients had higher levels of TMAO than controls, and these levels were associated with higher mortality risk (hazard ratio: 2.2 after adjustment for other risk factors). The mechanisms that link heart failure to TMAO levels, however, remain to be clarified. [240,241].

(d) Obesity, T2DM, and metabolic syndrome. Several studies have related obesity to an increased ratio of *Firmicutes* to *Bacteroidetes* [242,243]. In obese humans, there is decreased abundance in *Bacteroidetes* compared to lean individuals, and weight loss in these individuals increased their population. Overweight children also show an increased relative abundance of *Enterobacteriaceae* compared to children with normal BMI [244], while in human and animal models, there has been noticed an increased population of *Bifidobacteria* in obese subjects. Nevertheless, data about which specific species promote or prevent obesity are still conflicting. An increased population of *Firmicutes*, especially those of the *Ruminococcaceae* family, had a favorable impact on insulin sensitivity, plasma acetate and triglycerides, and was associated with positive changes in markers of brown adipocytes in subcutaneous (but not in visceral) fat in a study with diabetic, morbidly obese women and men who underwent elective gastric-bypass surgery [245]. After one year’s adherence to a Mediterranean Diet or an LFHC diet, the abundance of the *Roseburia* genus and *F*. *prausnitzii*, respectively, led to less insulin resistance in obese subjects [246]. A study by Scwiertz et al., which examined the fecal microbiota in normal, overweight, and obese subjects, showed that SCFAs, especially propionate, were increased in the feces of overweight and obese subjects [247]. A 24-week study involving 60 overweight adults where propionate was specifically delivered to the colon through a novel inulin-propionate ester stimulated the release of anorexigenic peptide YY (PYY) and GLP-1 from colonic cells, leading to eventual weight loss [248]. Similar to this result, other studies have shown an association between T2DM and decreased population of butyrate-producing bacteria and an increased abundance of *Lactobacillus* spp. [249].

(e) Hypertension. Few studies have directly linked the composition of gut microbiota to hypertension, mainly in animal models. Yang et al., showed an increased *Firmicutes*/*Bacteroidetes* ratio in hypertensive rats [250]. SCFAs could also play a role through the GPR pathways that impact renin secretion [251]. A recent meta-analysis of nine trials showed that the consumption of probiotics significantly changed systolic BP by −3.56 mmHg and diastolic BP by −2.38 mmHg, changes that were more profound in hypertensive subjects and when the probiotic intervention included many different species and lasted at least eight weeks [252]. However, many further studies are needed to elucidate the association between hypertension and microbiota.

### 7.3. A Novel Therapeutic Target?

The relationships between gut microbiota, diet, and cardiovascular disease imply that any intervention in the composition of the microbiome could be considered a therapeutic prospect. Currently, diet intervention is the main proposed regimen [253]. The exact dietary changes, however, that need to take place are difficult to be determined. As we mentioned above, even short changes in diet can dramatically affect the composition of the microbiota; the switch between plant- and meat-based diets or the addition of more than 30 g per day of specific fibers in daily dietary regimen, even for 10 days, results in shifts in microbiota composition and function in just over 1–2 days [254]. Contrary to these rapid dynamics, a 10-day feeding study in 10 people did not modulate the major characteristics of their microbiota, implying that long-term dietary habits also play a fundamental role in the synthesis of gut microbiome [255]. Even more intriguingly, a specific change in diet can have variable effects on different people due to the distinct combination of each individual’s gut microbial species, as molecular analyses of recent decades, including The Human Microbiome Project, have made evident [256]. The use of probiotics and prebiotics is on the increase [257,258]. However, changes in microbial populations are often relatively small, and generally persist only for as long as the period of the intervention. The use of antibiotics to change intestinal microflora is not widely accepted, as it is considered to do more harm than good, given the adverse effects that drugs have and the obvious inability to be administered for prolonged periods. Fecal microbiota transplantation (FMT) is a promising new therapeutic procedure for cardiometabolic disorders. In a study where overweight patients were transferred microbiota from healthy controls, hepatic and peripheral insulin sensitivity were improved by more than 100% compared to subjects that were transferred microbiota from their own feces [259]. However, the danger of transferring also harmful endotoxins and other agents with this procedure imposes some limitations to this technique, and more studies are needed to assure its safety [260,261].

## 8. Conclusions

As cardiovascular diseases are on a constant rise, the role of diet as a mediator gathers more and more interest. Dietary manipulation can reduce cardiovascular disease by 50%. Despite some controversial data and the definite need for more studies to come, dietary patterns that combine a high fat intake with low carbohydrates are no longer out of the question. Different kinds of fatty acids act differently in terms of atherosclerosis, inflammation, and oxidative stress. There is no doubt that ingestion of industrialized transunsaturated fats accelerates atherosclerosis and augments mortality. Saturated fats are also considered a potential macronutrient enemy for the heart and the endothelium while reducing them can mitigate CVD risk. Polyunsaturated fats and especially n-3 PUFAs seem to possess the most favorable potential. The role of the Mediterranean Diet in preventing CVD has been highlighted in recent years by many studies, and it is attributed to the combination of foods and micronutrients that this diet entails. These facts, combined with the new data supporting that different strains in the human gut microbiome lead to different metabolic responses and have various effects on cardiometabolic disorders, make dietary interventions a promising and exciting prospect for the treatment of cardiovascular disease in the future. What is important to remember, however, is that we do not consume macro- or micronutrients solely, but we rather integrate them within a holistic lifestyle pattern, where moderation is probably the key to the best outcome. For these reasons, larger RCTs are needed to actually prove the best individualized dietary approach for each metabolic milieu.

## Abbreviations

CVD	cardiovascular disease
T2DM	type 2 diabetes mellitus
WHO	World Health Organization
ESC	European Society of Cardiology
AHA	American Heart Association
MACE	major adverse cardiovascular event
MI	myocardial infarction
LDL	low-density lipoprotein
HDL	high-density lipoprotein
ApoA1	apolipoprotein A1
ApoB	apolipoprotein B
ApoE	apolipoprotein E
LCHF	low-carb, high-fat
LFHC	low-fat, high-carb
HbA1c	hemoglobin A1c
SFA	saturated fatty acid
CLA	Conjugated Linoleic Acid
MUFA	monounsaturated fatty acid
PUFA	polyunsaturated fatty acid
GLP-1	glucagon-like peptide-1
ApoB-100	apolipoprotein B-100
VLDL	very low-density lipoprotein
DHA	Docosahexanoic acid
EPA	eicosapentanoic acid
SREBP-1c	sterol regulatory element-binding protein-1c
TAG	triacylglycerol
TC	total cholesterol
TG	triglycerides
FA	fatty acid
IL	interleukin
TNF-α	tumor necrosis factor-α
IFN-γ	interferon-γ
NF-κΒ	nuclear factor-κΒ
Med-Diet	Mediterranean Diet
FMD	flow-mediated dilatation
LV	left ventricle
MetS	metabolic syndrome
RCT	randomized controlled trial
DASH	Dietary Approaches to Stop Hypertension
EGCG	Epigallocatechin-3-gallate
GIP	Gastric inhibitory polypeptide
SGLT	sodium-glucose transport protein
MMP-9	metallopeptidase 9
SIRT-1	sirtuin-1
eNOS	endothelial nitric oxide synthase
Nrf2	nuclear factor-like 2
ICAM-1	intercellular adhesion molecule-1
VCAM	vascular cell adhesion molecule-1
NADPH	nicotinamide adenine dinucleotide phosphate
NO	nitric oxide
PPAR-γ	peroxisome proliferator-activated receptor-γ
ABCA1	ATP-binding cassette transporter
LKB1	liver kinase B1
AMPK	AMP-activated protein kinase
MLCK	myosin light chain kinase
p-MLC	phosphorylated myosin light chain.
VSMC	vascular smooth muscle cells
GLUT-4	glucose transporter type 4
AMP	adenosine monophosphate
TLR4	toll-like receptor 4
ox-LDL	oxidized LDL
